# The Association of Sjögren Syndrome and Autoimmune Thyroid Disorders

**DOI:** 10.3389/fendo.2018.00121

**Published:** 2018-04-03

**Authors:** Chiara Baldini, Francesco Ferro, Marta Mosca, Poupak Fallahi, Alessandro Antonelli

**Affiliations:** ^1^Rheumatology Unit, University of Pisa, Pisa, Italy; ^2^Department of Clinical and Experimental Medicine, University of Pisa, Pisa, Italy

**Keywords:** Sjögren’s syndrome, autoimmune thyroid diseases, non-Hodgkin’s lymphoma, comorbidities, pathogenesis

## Abstract

Sjögren’s syndrome (SS) and autoimmune thyroid diseases (AITD) may frequently coexist in clinical practice, resulting in a complex overlapping disorder that represents a particular example of the expression of heterogeneity in patients with autoimmune disorders. Objective of this review was to describe the prevalence of the SS–AITD association in the most recent literature, exploring in particular to what extent the presence of AITD might influence the clinical expression of SS and *vice versa*. Moreover, we summarized some of the proposed genetic, biologic, and molecular mechanisms implied in the pathogenesis of AITD–SS association. Finally, we explored risk factors for lymphoma development in both AITD and SS. We performed a Medline search of English language articles published in the PubMed database in order to provide a critical overview of the recent literature on pathogenesis and clinical features of AITD–SS overlapping disease. All the articles were critically analyzed to select the most relevant contributions.

## Introduction

Sjögren’s syndrome (SS) is a complex and heterogeneous autoimmune disease that frequently co-occurs with both organ-specific and non-organ specific, systemic rheumatic diseases (i.e., rheumatoid arthritis, systemic lupus erythematosus, and systemic sclerosis) offering a unique opportunity to investigate pathogenesis, long-term-evolution, and outcome of different autoimmune phenotypes and subsets (1–5).

The association of SS and autoimmune thyroid diseases (AITD) has been largely documented, suggesting that AITD could be overrepresented in patient with SS with respect to general population and *vice versa* (6). Interestingly, Rojas-Villarraga et al. (7) analyzing prevalence of multiple autoimmune syndromes in 1,083 patients belonging to four autoimmune disease cohorts described AITD and SS as the most frequent coexisting autoimmune disorders in single patients. Overall, then, it has been hypothesized that common genetic, immunologic, and biologic factors may be implied in SS and AITD, leading to the coexistence of these two conditions (8). Moreover, both SS and AITD may lead to the development of non-Hodgkin’s lymphoma (NHL) highlighting the relationship between similar autoimmunity pathways and lymphoproliferation (9, 10). Although the majority of data support an increased prevalence of AITD in SS patients, some authors have suggested that this observation might be related to the age and sex distribution of SS and AITD rather than representing a true association. However, literature data are difficult to compare due to the fact that different SS classification criteria and AITD definitions have been utilized over the years, making it difficult to define the exact prevalence of SS–AITD association (11, 12).

In this review, we aimed at describing the frequency of the SS–AITD association in the most recent literature, exploring, in particular, to what extent the presence of AITD may influence the clinical expression of SS. Moreover, we will summarize some of the proposed pathogenetic mechanisms for the coincidence of SS and AITD and the common biologic factors involved into lymphoproliferative complications.

### Prevalence of SS in AITD: A Clinical Spectrum Ranging From Isolated Sicca Symptoms to Full-Blown SS

Autoimmune thyroid diseases, namely, Hashimoto’s thyroiditis (HT) and Graves’ disease (GD), are organ-specific autoimmune disorders, essentially resulting from a T cell-mediated immune attack of the thyroid resulting into lymphocytic infiltration of the thyroid parenchyma (13). The association between AITD and other organ specific (polyglandular autoimmune syndromes), or systemic rheumatic disorders has been widely described. SS is perhaps the most frequently rheumatic autoimmune disease associated with AITD, and particularly with HT. Several authors have reported that a full-blown SS could be 10 times higher in AITD than in the general population (14). In fact, the prevalence of SS in AITD has been assessed by several heterogeneous studies over the time and apparently varies from 3% up to 32% (15–17).

In 1992, Warfvinge et al. (18), analyzing different degrees of morphologic and functional salivary gland changes in AITD, found that 11 patients out of 19 presented xerostomia, a compromised unstimulated salivary flow and alterations of their lower lip biopsy, and/or parotid scintigraphy. Six of them presented a true SS. Similarly, Tektonidou (16) in a cohort of 58 patients with antinuclear antibodies positive AITD found that 9% fulfilled the criteria for SS. These data on small cohorts were confirmed also in large population studies over the years. For example, Biro et al. (19) evaluating the prevalence of SS in 426 patients with HT or GD found that SS had a prevalence of 17% in HT and of 5% in GD. Similarly, Lu et al. (20) observed that the risk of SS in patients with thyroiditis was 3.6 times higher than in individuals without thyroiditis.

The spectrum of sicca symptoms in patients with AITD that do not fulfill the criteria for SS is even more common. More specifically, Coll et al. (21) in a cohort of 176 patients with AITD found that 19 of 52 (37%) patients presented isolated xerostomia and 39/170 (23%) isolated keratoconjunctivitis sicca. Other authors described an isolated positivity for antinuclear antibodies in 25–55% of patients with AITD. The real implication of antinuclear antibodies positivity in AITD patients has to be clarified, but it is generally believed that they may reflect polyclonal activation and antibody production that over time may trigger the development of concomitant systemic autoimmune diseases including SS (22). In clinical settings, patients with AITD and a positivity for antinuclear antibodies should then be monitored more closely for the development of SS during the follow-up.

Overall, the available literature suggests that both a full blown SS and “incomplete” subsets of SS may be quite common among patients with AITD. Among these incomplete forms, an important differential diagnosis is represented by patients with sicca symptoms, affected by fibromyalgia. Patients with fibromyalgia often present dry eyes and dry mouth and noteworthy, they may present AITD or a positivity for thyroid auto antibodies as well. In the study by Mavragani et al. (23), fibromyalgia patients with sicca symptoms presented thyroid auto antibodies with a prevalence of 60%. Besides fibromyalgia, a number of other systemic disorders associated with AITD also involves salivary glands and should be considered as well in the differential diagnosis. Infectious diseases like hepatitis C virus and Ebstein B virus infections are among the major mimickers of SS in AITD patients, especially due to viral lymphotropism and epithelial tropism (24, 25). Immune disorders including sarcoidosis and IgG4 disease may be considered as well. The latter represents a novel entity characterized by high serum IgG4 and IgG4 plasma cell-mediated fibro-inflammatory lesions that may involve not only thyroid but also lung, pancreas, kidney, and salivary and lacrymal glands (26). More specifically, IgG4 has been linked to four types of thyroid diseases including: Riedel’s thyroiditis, fibrosing variant of Hashimoto’s thyroiditis, IgG4-related Hashimoto’s thyroiditis, and GD with elevated IgG4 levels (27). The pathogenetic role of IgG4 in these IgG4-related thyroid diseases, however, still remains poorly understood. Those patients may often present with an SS-like subacute diffuse enlargement of lacrymal and salivary glands and sicca symptoms. A minor salivary gland biopsy is often necessary to distinguish IgG4 disease from SS (28, 29).

### Prevalence of AITD in SS: A Specific Subset of the Disease?

Several uncontrolled studies have described the presence of AITD in SS with frequencies ranging between 10 and 30% (8). Table 1 summarizes the most important studies reporting frequency of AITD in SS. The differences observed among these studies might be linked to the different ethnic origin of the patients or to the diagnostic criteria adopted for both SS and HT classification.

**Table 1:** Prevalence of AITD in Sjögren’s syndrome.

Reference	Year	No. PTs	Prevalence (%)			
			HT	GD	Anti-TPO	Anti-Tg
Karsh et al. (30)	1980	24	nd	nd	41.6	20.8
Kelly et al. (31)	1991	100	14	nd	40	nd
Hansen et al. (32)	1991	28	18	nd	36	nd
Bouanani et al. (33)	1991	26	11.5	nd	nd	100
Foster et al. (34)	1993	42	nd	nd	36	36
Perez et al. (35)	1995	33	24	6	45	18
Punzi et al. (36)	1996	121	7	nd	17.6	13.4
Davidson et al. (37)	1999	74	nd	nd	22.8	nd
Ramos Casals et al. (38)	2000	160	20	1.25	15.6	12.5
D’Arbonneau et al. (39)	2003	137	14.6	0.7	10.9	2.9
Tunc et al. (40)	2004	53	4	nd	9	9
Lazarus and Isenberg (41)	2005	114	16	1.8	nd	nd
Biro et al. (19)	2006	400	7	3	nd	nd
Mavragani et al. (11)	2009	54	nd	nd	20.4	18.5
Zeher et al. (42)	2009	479	6.3	3.8	nd	nd
Caramaschi et al. (43)	2013	100	27	nd	nd	nd

*nd, not done; HT, Hashimoto’s thyroiditis; GD, Graves’ disease; Anti-TPO, anti thyroperoxidase antibodies; anti-Tg, antithyroglobulin antibodies, PTs, patients*.

As far as the clinical manifestation of AITD in SS, three studies are particularly relevant. In the study by Lazarus and Isenberg (41), 16% of the SS patients developed AITD. The most common clinical manifestation of AITD in SS was hypothyroidism, even though subclinical AITD was probably more common. In the vast majority of the cases, hypothyroidism had been diagnosed before the diagnosis of SS: similarly, Jara et al. (6) in a cohort of 160 primary SS patients found evidence of thyroid disease in 36% of patients: 20% were diagnosed as AITD and 16% were diagnosed as non-AITD. The clinical pattern in more than half of primary SS patients was subclinical hypothyroidism. Finally, in the study by Zeher et al. (42) 479 patients with pSS were investigated with regard to several types of thyroid disease. Overall, thyroid dysfunction was found in 95 patients (21.25%). Apparently, the diagnosis of HT and GD may be present either before or after the onset of SS but, differently from the study by Lazarus and Isenberg (41), both HT and GD were more likely to follow SS. The authors found that in 50% of the cases SS preceded HT by 5.5 years. Interestingly, D’Arbonneau et al. (39) showed that the presence of thyroid-related autoantibodies represented a risk factor for development of AITD during follow-up.

Finally, regarding the clinical impact of AITD on the clinical course, literature data are quite heterogeneous especially due to different control groups that have been included (i.e., SS–AITD versus SS without AITD; SS–AITD versus AITD) and to the classification criteria adopted to define SS. However, Caramaschi et al. (43) demonstrated that the association of AITD in patients suffering from SS defined a subset of patients with milder disease and normal complement 4 levels. More specifically, patients with AITD–SS had less evidence of cryoglobulins, palpable purpura, peripheral neuropathy, and lymphoma. This observation has been confirmed by several additional studies. However, it is nowadays widely accepted that AITD–SS patients are at a greater risk of developing additional autoimmune diseases such as autoimmune liver diseases, inflammatory bowel diseases, and systemic lupus erythematosus and should be closely monitored over the follow-up due to their tendency to present widespread disorders of their immune systems.

### SS and AITD: Common Pathogenetic Mechanisms From Epithelitis to Non-Hodgkin Lymphoma

A large amount of data have highlighted that AITD and SS can be considered as pathogenetically correlated. First of all, these two disorders are characterized by similar histological features with a tissue infiltrate that consists primarily of CD4+ T lymphocytes and the possible formation of germinal center-like structures unrevealing B cell activation (8). Second, from a genetic point of view, the two conditions present a similar background with thyroid and epithelial cells expressing the same HLA molecules class II: HLA-B8 and HLA-DR3 (8). In particular, HLA-B8 and -DR3 haplotypes have been reported with a higher frequency in both primary SS and AITD whereas cytotoxic T lymphocytic antigen 4 gene polymorphisms have been reported more frequently in patients with AITD and other autoimmune diseases including rheumatoid arthritis (15).

A third point to take into account is the existence of animal models that spontaneously develop both SS and AITD. Thyroiditis and SS in NOD.H-2h4 mice are chronic autoimmune diseases that develop relatively early in life and persist for the life of the animal making them an excellent model to test therapeutic protocols over a long period of time (44).

Another point reinforcing the pathogenetical link between AITD and SS is represented by the crucial role of the epithelial cells in orchestrating the tissue inflammation and the involvement of several regulatory chemokines, such as the IFN-γ-inducible protein 10 (IP-10/CXCL10) in initiating and perpetuating the autoimmune process. CXCL10 exerts its function through binding to chemokine (C-X-C motif) receptor 3 (CXCR3), and its production is stimulated by IFN-γ and TNF-α, whose production in turn is provided by T helper 1 cells (Th1) (45).

In AITD, it has been postulated that CXCL10 could be a marker of a stronger and more aggressive inflammatory response, subsequently leading to thyroid destruction and hypothyroidism (45). Similarly, epithelial cells from SS patients apparently produce CXCL9 and CXCL10 as well, while most of the CD3+ lymphocytes in periductal foci express CXCR3, thus contributing to salivary gland damage (46).

Another cytokine that exerts a fundamental role in the pathogenesis of both AITD and SS is B-cell activating factor that seems to be especially important for the survival of autoreactive B cells (47). Serum B-cell activating factor concentrations were described as significantly higher in GD and correlated with serum antithyroglobulin antibodies (48). B-cell activating factor has been also correlated to disease activity and severity in SS exerting a key role in lymphoproliferative complications (49–52).

Not surprisingly, both AITD and SS may evolve into B-cell NHLs of salivary glands and thyroid, with a prevalence of 0.5 and 5%, respectively (9). Intriguingly cases of patients with AITD, SS, and NHL have been described even if the vast majority of the studies seem to support the evidence that AITD–SS patients have a lower risk of developing NHL compared to SS patients without AITD (53–56). It remains a matter of debate whether the presence of SS in AITD patients may increase the risk of thyroid lymphoma in AITD patients.

The striking association between SS, AITD, and NHL has provided valuable insights into the relationship between autoimmunity and lymphoproliferation. In this multistep process, key players are represented by chronic inflammation and sustained antigenic stimulation that are apparently able to promote B-cell activation and proliferation. Immune deregulation, moreover, sometimes promotes B cell proliferation mediated by infectious agents (i.e., suppression/dysregulation of T-cells leading to EBV-driven B-cell proliferation) (57, 58). Ultimately, SS and AITD seem to be characterized by a wide spectrum of genetic and molecular abnormalities that culminate in uncontrolled B-cell activation, proliferation, and neoplastic transformation.

### Conclusion

The coexistence of SS and AITD occurs frequently in clinical practice probably due to common pathogenetic mechanisms shared by these two conditions. From a practical point of view, it is important to screen patients with SS for AITD and *vice versa* because the presence of the two disorders may influence patients’ clinical presentation and long-term outcome. It is widely accepted that AITD–SS patients may have a milder phenotype of SS with a lower risk for lymphoma development. However, it remains unclear whether a concomitant diagnosis of SS may increase or not the risk for thyroid lymphoproliferative complications in AITD patients. Intriguingly, AITD–SS patients over the follow up frequently present additional autoimmune diseases and should be closely monitored especially for liver autoimmunity. Further studies are warranted to explore genetic, biologic, and molecular factors underlying the SS–AITD association in order to provide further insights for the comprehension of this complex and varied subset of autoimmunity.

## Author Contributions

CB, FF, MM, PF, and AA gave substantial contribution in the conception and design of the work, in the literature research and in writing the paper; gave the final approval of the version to be published; agreed to be accountable for all aspects of the work in ensuring that questions related to the accuracy or integrity of any part of the work are appropriately investigated and resolved. CB, AA, and PF revised it critically.

## Conflict of Interest Statement

The authors declare that the research was conducted in the absence of any commercial or financial relationships that could be construed as a potential conflict of interest. The reviewer DL and handling Editor declared their shared affiliation.
